# Effect of High-Density Lipoprotein Metabolic Pathway Gene Variations and Risk Factors on Neovascular Age-Related Macular Degeneration and Polypoidal Choroidal Vasculopathy in China

**DOI:** 10.1371/journal.pone.0143924

**Published:** 2015-12-01

**Authors:** Qingyu Meng, Lvzhen Huang, Yaoyao Sun, Yujing Bai, Bin Wang, Wenzhen Yu, Mingwei Zhao, Xiaoxin Li

**Affiliations:** 1 Peking University People’s Hospital, Ophthalmology Department, Beijing, China; 2 Key Laboratory of Vision Loss and Restoration, Ministry of Education, Beijing, China; 3 Beijing Key Laboratory of Diagnosis and Therapy of Retinal and Choroid Diseases, Beijing, China; Univeristy of Miami, UNITED STATES

## Abstract

**Purpose:**

To investigate the effect of genetic variants in the high-density lipoprotein (HDL) metabolic pathway and risk factors on neovascular age-related macular degeneration (nAMD) and polypoidal choroidal vasculopathy (PCV) in China.

**Methods:**

A total of 742 Chinese subjects, including 221 controls, 230 cases with nAMD, and 291 cases with PCV, were included in the present study. Five single nucleotide polymorphisms (SNPs) from three genes in the HDL metabolic pathway (HDLMP) including cholesteryl ester transfer protein (*CETP*), hepatic lipase (*LIPC*) and lipoprotein lipase (*LPL*) were genotyped in all study subjects with matrix-assisted laser desorption/ionization time-of-flight mass spectrometry (MALDI-TOF-MS). Risk factors including gender, hypertension, hyperlipidemia, diabetes mellitus, and coronary artery disease were identified. Chi-square tests or Fisher’s exact tests were applied to discover associations between SNPs and risk factors for PCV and nAMD. Gene-gene interactions and gene-environment interactions were evaluated by the multifactor-dimensionality reduction (MDR) method.

**Results:**

*CETP* rs3764261 were significantly associated with an increased risk for PCV (odds ratio (OR) = 1.444, P = 0.0247). *LIPC* rs1532085 conferred an increased risk for PCV (OR = 1.393, P = 0.0094). We found no association between PCV and *LPL* rs12678919, *LIPC* rs10468017 or *CETP* rs173539. No association was found between five SNPs with nAMD. Regarding risk factors, females were found to have significantly decreased risks for both PCV and nAMD (P = 0.006 and 0.001, respectively). Coronary artery disease (CAD) was a risk factor in PCV patients but played a protective role in nAMD patients. Hyperlipidemia was associated with PCV but not with nAMD. Neither hypertension nor diabetes mellitus was associated with PCV or nAMD. The MDR analysis revealed that a three-locus model with rs12678919, rs1532085, and gender was the best model for nAMD, while a five-locus model consisting of rs10468017, rs3764261, rs1532085, gender, and hyperlipidemia was best for PCV.

**Conclusion:**

Our large-sample study suggested that *CETP* rs3764261 conferred an increased risk for PCV. We also first found the association between rs1532085 and PCV. The result of present study also showed that gender and CAD are associated with PCV and nAMD. Significant association was found between hyperlipidemia and PCV but not nAMD.

## Introduction

Age-related macular degeneration (AMD) is the major cause of vision loss in the elderly in developed countries [1]. Advanced AMD consists of two forms: geographic atrophy (GA) of the retinal pigment epithelium (RPE) with overlying photoreceptors (called advanced “dry” AMD) and choroidal neovascularization (CNV, called “wet” AMD or neovascular AMD, nAMD). Polypoidal choroidal vasculopathy (PCV) is another important form of maculopathy found in a significant proportion of elderly Chinese and Japanese patients, and it manifests as orange-reddish nodules or polyp-like structures at the posterior pole [2]. Indocyanine green angiography (ICGA) can be used to definitively diagnose PCV. It has been observed that PCV and nAMD share some clinical and pathological features, such as demographics [3], risk factors [4], and manifestation [3]; however, whether PCV is a variant of CNV or is a distinctive disease characterized by an inner choroidal vascular abnormality also remains debatable.

Recently, two genome-wide association studies (GWAS) identified that genes in the high-density lipoprotein (HDL) cholesterol metabolic pathway were associated with AMD [5,6]. Some studies have identified genetic loci, such as rs10468017 near hepatic lipase (*LIPC*) and rs12678919 near lipoprotein lipase (*LPL*), were associated with late AMD [7–10], while other studies have found no association with nAMD [11,12]. However, results of replication studies in different cohorts on the reported HDL cholesterol metabolism genes, including the *hepatic lipase* (*LIPC*), *cholesterylester transfer protein* (*CETP*), and *lipoprotein lipase* (*LPL*) genes were inconsistent[13–15]. It is well documented that genotype-phenotype associations may vary in different populations; therefore, further investigations that attempt to replicate these associations in the Asian population.

As we know that, besides genetic factors, environment risk factors and lifestyles also participated in the development of AMD and PCV. However, due to the different ethnicities and lifestyles of individuals, the evidence and strength of such associations are widely variable [16–18]. Thus, we performed this study to investigate the effect between gene variations in the HDL cholesterol metabolic pathway and risk factors on nAMD and PCV in China.

## Materials and Methods

### Subjects

A total of 742 unrelated Chinese subjects were studied in this case-control cohort; specifically, 230 patients had nAMD, 291 patients had PCV, and 221 individuals without age-related maculopathy (ARM) were studied as controls. The study participants were recruited from the Department of Ophthalmology at the Peking University People’s Hospital, and the study was approved by the Ethical Committee of Peking University People’s Hospital. An informed consent process was established following the guidelines of the Helsinki Declaration, and all subjects signed consent forms. All subjects underwent a comprehensive ophthalmic examination, including visual acuity measurements, slit-lamp biomicroscopy, and dilated fundus examinations, performed by a retinal specialist. All cases with AMD and PCV underwent fluorescein angiography, optical coherence tomography (OCT), and indocyanine green angiograms with HRA2 (Heidelberg Engineering, Heidelberg, Germany). The diagnosis of AMD or ARM was defined in accordance with the International Classification System for ARM [19]. A PCV diagnosis was based on indocyanine green angiography (ICGA) results that showed a branching vascular network terminating in aneurysmal enlargements, which typify polypoidal lesions. Exclusion criteria included any eye with any other macular abnormalities, such as pathological myopia, idiopathic choroidal neovascularization (CNV), presumed ocular histoplasmosis, angioid streaks, and any other secondary CNV. Normal controls were defined as having no clinical evidence of AMD or PCV in either eye or any other eye diseases, excluding mild age-related cataracts. Subjects with severe cataracts were excluded from the study. Information on hypertension, diabetes mellitus (DM), hyperlipidemia, and coronary artery disease (CAD) was obtained by a questionnaire.

### Single Nucleotide Polymorphism Selection and Genetic Analysis

Five single nucleotide polymorphisms (SNPs) in three genes associated with AMD or PCV were selected according to the literature, which were rs10468017 in *LIPC*[5,6,20]; rs3764261 in *CETP*[5,6]; rs12678919 near *LPL*[5,6,20]. We also included *LIPC* rs1532085 and *CETP* rs173539, which regulated gene expression and showed impact on HDL levels[21,22]. Blood samples were collected from all participants and stored at -80°C before DNA extraction. Genomic DNA was extracted from venous blood leukocytes using a genomic extraction kit (Beijing eBios Biotechnology Co., Ltd.), and genotyping was performed by matrix-assisted laser desorption/ionization time-of-flight mass spectrometry (MALDI-TOF-MS), as previously described [23]. Briefly, approximately 30 ng of genomic DNA was used to genotype each sample. The DNA samples were amplified, and the PCR products were used for locus-specific single-base extension reactions. The resulting products were desalted and transferred to a 384 SpectroCHIP array. Allele detection was performed using MALDI-TOF-MS. The mass spectrograms were analyzed using MassARRAY Typer software version 4.0 (Sequenom, San Diego, CA, USA).

### Statistical Analysis

The data were analyzed using SPSS (version 16.0; SPSS Science, Chicago, IL). All of the identified polymorphisms were assessed for Hardy-Weinberg equilibrium using chi-square tests. Single-marker association analyses were performed using chi-square tests or Fisher’s exact tests under various genetic models. Risk factors, including gender, hypertension, DM, hyperlipidemia, and CAD, were independently screened for associations between nAMD and PCV by applying chi-square tests or Fisher’s exact tests. Logistic regression models were used to calculate the odds ratio (OR) and 95% confidence interval (CI) of nAMD or PCV, with case groups compared with the control group. Values of P<0.05 were considered statistically significant. Joint effect analysis for of the significant SNPs identified from the main effects analysis (P<0.05) was also performed to evaluated potential dose-response effects by adding up the number of alleles. To assess gene-gene interaction and gene-environment interactions, we employed the non-parametric multifactor dimensionality reduction (MDR) method [24,25]. We selected the model with the highest test accuracy and cross-validation consistency (CVC). We further evaluated the model using permutation testing 10,000 times, and we repeated the MDR analysis on each randomized dataset. This analysis was performed using nonparametric MDR software (version 2.0 alpha, www.multifactordimensionalityreduction.org).

## Results

A total of 742 subjects participated in the study, including 221 control subjects (mean age ± standard deviation [SD], 67.2±9.6 years; 52.0% females), 230 cases with nAMD (mean age ± SD, 69.3±8.8 years; 36.5% females) in one or both eyes, and 291 cases with PCV (mean age ± SD, 66.6±9.6 years; 39.9% females) in at least one eye. The genders and ages of the controls and cases are provided in Table 1.

**Table 1:** Demographic Distribution of the study subjects.

	Controls (n = 221)	nAMD(n = 230)	PCV(n = 291)
Females, n (%)	115(52.0)	84(36.5)	116(39.9)
Age* range (Years)	45–96	50–90	42–87
Mean age ± SD** (Years)	67.2±9.6	69.3±8.8	66.6±9.6

* Age of presentation.

** SD, standard deviation

nAMD: neovascular age-related macular degeneration; PCV: polypoidal choroidal vasculopathy.

### Association between individual SNPs with nAMD and PCV

The genotype frequencies for patients and controls are shown in Table 2. No SNPs showed any significant deviation from Hardy-Weinberg equilibrium (P>0.05), except *CETP* rs173539 in any groups and rs10468017 in nAMD group (P<0.05) (Shown in S1 Table).

**Table 2:** Association between individual SNP and risk factors with nAMD and PCV risk.

		Control	PCV	nAMD	PCV vs Control		nAMD vs Control	
SNP	Risk Allele	A1/A2	A1/A2	A1/A2	OR (95%CI)	P-value	OR (95%CI)	P-value
*LPL* rs12678919	G	44/398	53/529	40/420	0.906 (0.595,1.38)	0.646	0.8615 (0.5495,1.351)	0.515
*LIPC* rs10468017	T	79/363	96/546	66/394	0.908(0.654,1.259)	0.562	0.7697 (0.5388,1.1)	0.150
*LIPC* rs1532085	A	209/233	228/354	192/268	1.393(1.084,1.789)	**0.0094**	0.7987 (0.6139,1.039)	0.0938
*CETP* rs173539	T	26/416	35/547	40/240	1.024(0.607,1.728)	0.930	1.524 (0.9132,2.543)	0.105
*CETP* rs3764261	T	71/371	126/456	86/374	1.444(1.047,1.991)	**0.0247**	1.202(0.8505,1.697)	0.297
Female		115	116	84	0.611(0.429, 0.870)	**0.006**	0.530(0.364, 0.773)	**0.001**
Hypertension		143	183	134	0.924(0.642, 1.330)	0.672	0.761(0.520, 1.114)	0.160
Diabetes Mellitus		189	258	186	1.324(0.786, 2.229)	0.292	0.716(0.435, 1.178)	0.188
Hyperlipidemia		169	264	173	3.009(1.819, 4.997)	**0.000**	0.934(0.607, 1.438)	0.756
CAD		204	284	197	3.381(1.377,8.302)	**0.008**	0.497(0.268, 0.922)	**0.027**

nAMD: neovascular age-related macular degeneration; PCV: polypoidal choroidal vasculopathy; CAD: coronary artery disease. SNP: single nucleotide polymorphism.

Regarding PCV, the risk allele (T) of *CETP* rs3764261 showed a strong association with an increased risk of PCV (OR = 1.444, 95%CI 1.047–1.991, P = 0.0247), while *CETP* rs173539 showed no association with PCV (P = 0.930). The risk allele A of *LIPC* rs1532085 turned out to be a risk factor for PCV (OR = 1.393, 95%CI 1.084–1.789, P = 0.0094). No significant association was found between the PCV group and *LPL* rs1257891 and *LIPC* rs10468017. However, among nAMD patients, we found no association with 5SNPs (all p>0.5).

### Association between environmental factors and nAMD and PCV

In our study, we found that females had significantly decreased risks of both PCV and nAMD (P = 0.006 and 0.001, respectively). CAD showed a risk effect in PCV patients (OR = 3.381, 95%CI 1.377–8.302), but it played a protective role in nAMD patients (OR = 0.497, 95%CI 0.268–0.922). Our analysis revealed that hyperlipidemia was significantly associated with PCV (OR = 3.009, 95%CI 1.819–4.997, P = 0.000), but not nAMD. No association of hypertension and DM with PCV or nAMD was found.

### Association of the cumulative effects of the SNPs with nAMD and PCV

We also investigated potential dose-response and cumulative effects of the significant main-effect SNPs in a joint analysis. As shown in Table 3, the OR increased as the number of risk alleles for nAMD and PCV in individual patients increased (P = 0.024 and 0.048, respectively.).

**Table 3:** Association between the cumulative effects of the SNPs and nAMD and PCV risk.

No. of risk alleles	Control	PCV	nAMD	OR^a^(95%CI)	P^a^	OR^b^(95%CI)	P^b^
< = 1	37	23	18	Ref		Ref	
2	105	55	51	0.843(0.456, 1.557)	0.585	0.998(0.519, 1.922)	0.996
3	147	104	100	1.138(0.639, 2.028)	0.661	1.398(0.754, 2.594)	0.288
4	142	114	102	1.291(0.726, 2.297)	0.384	1.477(0.796, 2.739)	0.217
5	60	50	54	1.341(0.706, 2.547)	0.371	1.850(0.944, 3.625)	0.073
6	14	10	7	1.149(0.438, 3.013)	0.778	1.028(0.353, 2.990)	0.960
> = 7	9	15	14	2.681(1.010, 7.120)	0.048	3.198(1.166, 8.772)	0.024

ORa, odds ratio of PCV to control

ORb, odds ratio of AMD to control

nAMD: neovascular age-related macular degeneration; PCV: polypoidal choroidal vasculopathy.

### Gene-gene and gene-environment interactions

The five SNPs and environmental factors were included in the MDR analysis to assess their interaction. Table 4 summarizes the best joint risk factor interaction models obtained for PCV and nAMD. In models examining nAMD, the best overall model was the model assessing the joint effect of the three-locus model with rs12678919, rs1532085, and gender, yielding a perfect CVC and a test accuracy of 0.6074 (P = 0.0000). The second most predictive model was the single-locus model incorporating gender (CVC = 100, test accuracy = 0.5771, P = 0.0009). The combination of rs1532085 and gender was also predictive, with a CVC of 98 and a test accuracy of 0.5757 (P = 0.0018). Among the PCV models, we found that the five-locus model incorporating rs10468017, rs3764261, rs1532085, gender, and hyperlipidemia was the best model overall, with a perfect CVC and a test accuracy of 0.6195 (P = 0.0000), while the next most predictive model, a four-locus model incorporating rs10468017, rs1532085, gender, and hyperlipidemia, was nearly as predictive.

**Table 4:** Summary of Multifactor Dimensionality Reduction (MDR) results for interactions analysis on nAMD and PCV risk.

Factors	MDR models	Testing accuracy	CVC	*P*	OR	footnote
**nAMD risk**						
1	Gender	0.5771	100/100	0.0009	1.8594 (1.1057,3.1270)	*BETTER*
2	rs1532085, gender	0.5757	97/100	0.0018	2.1212 (1.2558,3.5832)	*BETTER*
3	rs12678919, rs1532085, gender	0.6074	100/100	0.0000	2.5561 (1.5034,4.3459)	*BEST*
4	rs12678919, rs173539, rs1532085, gender	0.5689	81/100	0.0060	2.9637 (1.7416,5.0435)	*BETTER*
5	rs12678919, rs3764261, rs1532085, gender, hypertension	0.5611	98/100	0.0018	3.9261 (2.2747,6.7765)	*BETTER*
**PCV risk**						
1	Hyperlipidemia	0.5580	98/100	0.0284	2.9376 (1.4407,5.9896)	*BETTER*
2	rs1532085, hyperlipidemia	0.5395	69/100	0.2421	2.9052 (1.6133,5.2317)	*X*
3	rs10468017, rs1532085, hyperlipidemia	0.5508	91/100	0.0666	2.6466 (1.6024,4.3712)	*BETTER*
4	rs10468017, rs1532085, gender, hyperlipidemia	0.6325	100/100	0.0000	3.6203 (2.1397,6.1254)	*BETTER*
5	rs10468017, rs3764261, rs1532085, gender, hyperlipidemia	0.6195	100/100	0.0000	4.4125 (2.6154,7.4443)	*BEST*

CVC—**Cross-validation consistency**

P-value based on 10 000 permutations

Best model with highest CVC and accuracy in bold.

nAMD: neovascular age-related macular degeneration; PCV: polypoidal choroidal vasculopathy.

## Discussion

HDL metabolic pathway genes, including *CETP*, *LIPC*, and *LPL*, are identified by genome-wide studies and are associated with AMD. CETP facilitates the transfer of triglycerides from very-low-density lipoproteins and low-density lipoproteins to HDL in exchange for cholesterol ester [26]. CETP transports cholesterol from peripheral tissues to the liver and regulates the concentration of HDL cholesterol. CETP was found to be located in the interphotoreceptor matrix in the monkey retina and transfers oxidized lipids from the outer segments of the photoreceptors and other membranes to HDL-like lipoprotein particles, which are then internalized by the RPE and excreted back into the circulation through Bruch’s membrane [27]. Therefore, a dysfunction in CETP may lead to the accumulation of oxidized lipids, which contributes to the development of nAMD and PCV. Nakata, I. et al found that rs3764261 was significantly associated with PCV among Japanese population[28]. There was a significant association between rs3764261 and PCV in our study, which was consistent with previous study. These results indicated that rs3764261 was susceptible for PCV in Eastern Asian population. Liu et al [10]reported that CETP was a susceptible gene for nAMD (OR = 1.89, *P* = 1.82×10^−4^). The association was also significant in the result of GWAS and EWAS by Cheng et al[29] in East Asian population (P = 1.66× 10^−12^). In the present study, the association between *CETP* rs3764261 and nAMD was in the same direction with these studies, although it did not reach a significant difference (OR = 1.202,P = 0.297). Several larger sample cohorts in Caucasian population also found a similar result that rs3764261 variant near *CETP* was associated with a non-significant increased risk of nAMD[12,20,30]. The two GWASs by Chen et al[6] in Japanese population and Nakata, I. et al[28] in the Caucasian population also indicted that *CETP* rs3764261 conferred increase risk for advanced AMD (OR = 1.19 and 1.12,respectively), but it did not reach the genome-wide significant (*P* = 7.4 × 10^−7^ and 1.41 × 10^−3^, respectively). Our results are generally consistent with theses previous studies. Larger sample studies are needed to verify the association between *CETP* rs3764261 and nAMD in Chinese population.

The hepatic lipase (*LIPC*) gene encodes hepatic triglyceride lipase, which has been shown to catalyze the hydrolysis of phospholipids, monoglycerides, diglycerides, triglycerides, and acyl-CoA thioesters, and it is a critical enzyme in HDL metabolism. In our study, we demonstrated for the first time that *LIPC* rs1532085 was associated with an increased risk for PCV. Rs1532085, located in the regulatory region of *LIPC*, is an eQTL of *LIPC and cis-regulates LIPC expression [31]. It has been associated with HDL-C, total cholesterol, and triglyceride levels [32,33]*. Furthermore, another gene-gene interaction study indicated that *LIPC*, especially the rs1532085 variant, might play a major role in the gene-gene interactions that regulate HDL-c levels [34]. Therefore, rs1532085 may participate in the development of PCV by regulating the HDL-C level.

*LIPC* rs10468017, located on chromosome 15 (15q21.3), has been studied with respect to its association with AMD. Several studies showed a decreased risk for AMD among Caucasians with the G allele of *LIPC* rs10468017[5,7]. In contrast, two studies showed no association between AMD and rs10468017 in the Chinese population [11,12]. Our results did not reveal a significant association between nAMD or PCV and the rs10468017 SNP, which is consistent with the two case-control studies.

Regarding the lipoprotein lipase (*LPL*) gene, it encodes LPL and has the dual functions of facilitating triglyceride hydrolysis and serving as a ligand/bridging factor for receptor-mediated lipoprotein uptake. It also plays an important role in HDL metabolism. Results regarding the association between *LPL* and AMD are not consistent. One GWAS [5] and three case-control studies [12,13,15] revealed a non-significant decreased risk for AMD among G allele carriers of the rs12678919 variant. However, another study [6] found a significantly increased risk for AMD among G carriers. Our results for nAMD are consistent with the former researches, as we showed that there was a non-significantly decreased risk for nAMD among risk allele (G) carriers (OR = 0.8615, P = 0.515). For PCV, no association was found with rs12678919 in a Japanese study[28], which was consistent with our results (P = 0.646).

Our results demonstrated that being female played a protective role in both PCV and nAMD patients, and this association was significant. This finding has been reported elsewhere, and it has also been found that PCV is more common among males than females among Asians [35]. However, several studies have reported that the wet form of AMD is more common in women than in men[36,37]. The reason for this discrepancy could result from the higher smoking rates by men. As we know that, smoking is an important consistent risk factor for nAMD. Previous study found that persons who smoked were more likely to have PCV or CNV secondary to AMD than non-smokers[38]. In China, cigarette consumption has increased substantially since the 1980s, almost exclusively in men. In particular, the higher prevalence of smoking in men in Asia, and the stronger associations of smoking with PCV and CNV secondary to AMD[39]. Therefore, the high smoking rate by men could be an important confounder. Furthermore, female had independently increased odds of perceived unmet health care needs than male, which made it easier for men to be diagnosed for nAMD[40]. Large cohorts and further research are needed to confirm the role of gender in the development of nAMD and PCV.

Previous studies have revealed lipid deposition in the Bruch membrane and soft drusen, and the quantity of lipid was higher in the macula than in the peripheral area of human eyes [41]. Therefore, researchers have explored the important role of lipids in the pathogenesis of AMD. However, no association was found between serum lipid levels (HDL-C and total cholesterol levels) and AMD over a 20-year period based on data from three population-based cohort studies, namely, the Beaver Dam Eye Study (BDES), Blue Mountains Eye Study (BMES), and Rotterdam Study (RS) [42]. Our results also did not show any significant associations between hyperlipidemia and nAMD. This finding suggests that the serum lipid level may not reflect the locally expressed lipid level in the retina. Moreover, it is not clear whether a high serum lipid level adversely regulates the expression of lipids in the retina. Zheng et al. used a high cholesterol diet and oral administrations of simvastatin to modulate serum cholesterol in mice. This study demonstrated that these treatments only modestly affected cholesterol content in the retina, but they had no significant effect on retinal expression of the major cholesterol- and vision-related genes [43]. Further research is needed to explore the relationship between lipid levels in the serum and the retina. Interestingly, we found a significant association between hyperlipidemia and PCV, which was previously rarely reported[44]. The finding of different associations of hyperlipidemia with PCV and nAMD suggests similar but not identical etiologies of the two diseases.

The possible association between CAD and AMD has previously been explored because they share a variety of risk factors, including smoking, hyperlipidemia, and chronic inflammatory processes at the site of pathogenesis [45–49]. However, the results from epidemiological studies and genetic analyses have been ambiguous. The meta-analysis by Usha Chakravarthy et al. revealed no significant association in the prospective cohort and the cross-sectional studies (RR 1.22, 95% CI 0.92–1.63 and OR 1.12; 95% CI 0.86–1.47), but a significant association was observed in the case-control studies, with an approximately two-fold increase in the probability of late AMD in individuals with cardiovascular disease (OR 2.20; 95% CI 1.48–3.26) [17]. In the Australian Heart Eye Study (AHES) [50], researchers reported that the severity of coronary stenosis and the presence of stenotic lesions of CAD were independently associated with early AMD but showed no associations with late AMD. However, an inverse association was reported between nAMD and cardiovascular disease requiring hospitalization in a large case-control study [51], which was in accordance with our results. We found that CAD conferred an increased risk for PCV (OR 3.381, 95%CI 1.377–8.302) and a decreased risk for nAMD (OR 0.497, 95%CI 0.262–0.992). Our results highlight the importance of monitoring individuals with evidence of CAD for signs of PCV. Furthermore, we speculated that if CAD patients get better as they receive treatment, their risk of nAMD would increase. Therefore, we recommend that patients with CAD who receive treatments also need to follow up for nAMD signs. Moreover, CAD patients have high morality rate. 3.9% major adverse cardiac events, including death, myocardial infarction, and late revascularization, occurred at about 2.4 year follow-up[52]. Survival effect could be a confound factor in the analysis.

In our study, we did not find associations of hypertension or DM with patient group. Our results are not consistent with those of a meta-analysis [17], which showed that hypertension and DM were moderately associated with late AMD. Patients with PCV were reported to have a higher prevalence of hypertension, diabetes mellitus compared with controls in Japanese population[4]. But, no association was found between hypertension or DM and PCV in China[53]. A possible explanation for this discrepancy is the low awareness of these two diseases in China. The prevalence of hypertension was 29.6%, but the awareness rate was less than 50% in a national survey [54]. In northeastern China, approximately 8.2% of participants were diagnosed with DM, and the awareness rate was 64.1% [55]. According to the epidemiology studies above, we speculated that the actual number of patients with these two diseases is larger than that in our study. Summary of the impact of genetic variants and risk factors on nAMD and PCV between previous reports and this study were shown in S2 Table.

To assess the cumulative effects of SNPs in our study, we performed a joint analysis and found that the OR of PCV and nAMD patients increased with the number of risk alleles, indicating that these genes may act independently in the pathogenesis of PCV and nAMD. We also performed MDR analysis to explore gene-environment interactions. The MDR method collapses high-dimensional genotype predictors (i.e., SNPs) into a single dimension and provides greater power to assess gene-gene and gene-environment interactions[56]. Regarding PCV patients, the best overall predictive model is the five-locus model incorporating rs10468017, rs3764261, rs1532085, gender, and hyperlipidemia, with perfect CVC and a high test accuracy, showing that the combination of *CETP*, *LIPC*, gender, and hyperlipidemia plays an important role in the development of PCV. Regarding nAMD, the combination of rs12678919, rs1532085, and gender is more predictive. The different models found to best predict PCV and nAMD indicate the different roles of the gene-environment interactions in the pathogenesis of these diseases. In our study, the testing accuracy of MDR analysis was approximately 0.6, which was insufficiently high. Therefore, to improve the testing accuracy, further studies using large samples are warranted to confirm these results.

There are several limitations of this study. One limitation is the sampled population. Because our study was conducted at a university hospital, which may have led to the inclusion of patients with more severe disorders compared with those in the general population. Second, smoking is not included in the risk factors analysis. Among environmental risk factors for AMD, active smoking is reported to be an important one. Overwhelming evidence showed that smokers have a greater prevalence of AMD than non-smokers in different ethnic population[38,44,57,58]. In non-smokers, people can also be exposed to the hazards of tobacco via passive smoking or secondhand smoking[59]. J C Khan et al[39] reported that passive smoking exposure was associated with an increased risk of AMD in non‐smokers in a case-control study. The prevalence of passive smoking is as high as 30% in China in 2010[60]. However, the duration of passive smoking of Chinese is hard to define, as smoking in public place and at homes is quite common in China, which may easily cause recall bias for participants. Moreover, the cessation period differs a lot from months to years in former smokers. The diversity smoking status made it difficult to analyze the accurate association between smoking and nAMD and PCV. Considering the aforementioned circumstances, we did not include smoking in this study. However, based on the results of previous large cohorts, we believe that smoking is a risk factor for nAMD and PCV. Moreover, in our study, we did not analyze the association with lipids level and patients. Up to now, the data we collected was not large enough to perform the analysis, and we will report our results on this subject in future. Finally, there is the potential for residual confounding effects from unmeasured or unknown parameters, which were not adjusted for in this study.

In conclusion, the result of present study suggested that *CETP* rs3764261 was associated with PCV, and conferred a non-significantly increased risk for nAMD. We also first found the association between rs1532085 and PCV. Our study found that gender and CAD are associated with nAMD and PCV and that hyperlipidemia is a risk factor for PCV. The risk factors we found take more attention to nAMD or PCV patients who have CAD or hyperlipidemia.

## Supporting Information

S1 Table Hardy-Weinberg equilibrium. (DOC)

S2 Table Comparison of impact of genetic variants and risk factors on nAMD and PCV between previous reports and this study. (DOCX)
