# Supplementary material for: Effect of High-Density Lipoprotein Metabolic Pathway Gene Variations and Risk Factors on Neovascular Age-Related Macular Degeneration and Polypoidal Choroidal Vasculopathy in China
Source: PLoS One. 2015 Dec 1;10(12):e0143924. doi: 10.1371/journal.pone.0143924 (PMC4666634; doi:10.1371/journal.pone.0143924)
Supplement: S1 Table — (DOC) [file pone.0143924.s001.doc]

**S1** Hardy-Weinberg equilibrium

| SNP | TEST | A1 | A2 | GENO | P-value |
| --- | --- | --- | --- | --- | --- |
| rs12678919 | Control | G | A | 2/40/179 | 0.8866 |
| rs10468017 | Control | T | C | 5/69/147 | 0.3452 |
| rs1532085 | Control | A | G | 52/105/64 | 0.4851 |
| rs173539 | Control | T | C | 13/0/208 | 4.752E-022 |
| rs3764261 | Control | T | G | 8/55/158 | 0.2517 |
| rs12678919 | nAMD | G | A | 2/36/192 | 0.8285 |
| rs10468017 | nAMD | T | C | 1/64/165 | 0.04508 |
| rs1532085 | nAMD | A | G | 36/120/74 | 02698 |
| rs173539 | nAMD | T | C | 20/0/210 | 4.35E-030 |
| rs3764261 | nAMD | T | G | 10/66/154 | 0.7518 |
| rs12678919 | PCV | G | A | 3/47/241 | 0.6777 |
| rs10468017 | PCV | T | C | 10/76/235 | 0.2155 |
| rs1532085 | PCV | A | G | 49/130/112 | 0.2856 |
| rs173539 | PCV | T | C | 17/1/274 | 4.34E-27 |
| rs3764261 | PCV | T | G | 12/102/177 | 0.5712 |

nAMD: neovascular age-related macular degeneration; PCV: polypoidal choroidal vasculopathy
