# Supplementary material for: Effect of High-Density Lipoprotein Metabolic Pathway Gene Variations and Risk Factors on Neovascular Age-Related Macular Degeneration and Polypoidal Choroidal Vasculopathy in China
Source: PLoS One. 2015 Dec 1;10(12):e0143924. doi: 10.1371/journal.pone.0143924 (PMC4666634; doi:10.1371/journal.pone.0143924)
Supplement: S2 Table — (DOCX) [file pone.0143924.s002.docx]

Supplement Table 2 Comparison of impact of genetic variants and risk factors on nAMD and PCV between previous reports and this study

| Genetic variants or Risk Factors | nAMD | | PCV | |
| --- | --- | --- | --- | --- |
|  | Our results | Previous Reports | Our results | Previous Reports |
| *CETP* rs3764261 | Non-significant increased | Increased: [10,12, 20,29,30] | Increased | Increased: [10,28] |
| *CETP* rs173539 | No association | None | No association | None |
| *LIPC* rs1532085 | No association | None | Increased | None |
| *LIPC* rs10468017 | No association | Decreased：[5,7]  No association: [11,12] | No association | None |
| *LPL* rs12678919 | Non-significant decreased | Non-significant decreased: [5, 12, 13, 15]  Increased: [6] | No association | No association: [28] |
| Gender (Female) | Decreased | Increased: [36] | Decreased | Decreased: [35] |
| Hyperlipidemia | No association | No association: [42] | No association | No association: [44] |
| CAD | Decreased | Increased：[17], [50]  Decreased: [51] | Increased | NONE |
| Hypertension | No association | Increased：[17] | No association | Increased: [4]  No association: [51] |
| Diabetes Mellitus | No association | Increased：[17] | No association | Increased: [4]  No association: [51] |

nAMD: neovascular age-related macular degeneration; PCV: polypoidal choroidal vasculopathy; CAD: coronary artery disease.
